# Allostatic load as a framework for understanding early-onset colorectal cancer: current biomarker evidence and future directions

**DOI:** 10.3389/fonc.2026.1823995

**Published:** 2026-09-08

**Authors:** Efosa Enoma, Atuahene Adu-Gyamfi, Jianna Carter, Sarah Wu, Barbara Nemesure, Jennie L. Williams

**Affiliations:** 1 Stony Brook Medicine, Stony Brook, NY, United States; 2 Stony Brook University, Stony Brook, NY, United States; 3 Ward Melville High School, East Setauket, NY, United States; 4 Department of Family, Population and Preventive Medicine, Stony Brook Medicine, Stony Brook, NY, United States; 5 Stony Brook Cancer Center, Stony Brook, NY, United States

**Keywords:** allostatic load, early-onset colorectal cancer, epigenetics, lifestyle, microbiome

## Abstract

**Introduction:**

Colorectal cancer incidence rates have been on the decline over the past 50 years due to increased screening and diagnostic tools. Over the past 32 years (since 1994), there has been a rise in colorectal cancer for those under 50 years old. This trend has been designated as early-onset colorectal cancer (EOCRC) and is in direct opposition to the decline seen in those over 50 years of age; designated as late onset colorectal cancer (LOCRC). The rise in EOCRC has been attributed to various factors such as environmental, modern diet and lifestyle.

**Methods:**

The paper includes a comprehensive search of both cohort and case-control studies that were designed to investigate the association between EOCRC and allostatic load (AL) using two databases: MEDLINE PubMed and Stony Brook University Libraries Database.

**Results:**

12 studies were included in this paper. These papers varied on how many used biomarkers that are measured in allostatic load. Out of the 12 studies, only 3 had direct measurements of AL such as metabolic and cardiovascular, 9 measured indirect biomarkers such as inflammatory pathways and epigenetics.

**Conclusion:**

The purpose of this paper is to change the current paradigm focus of the causation of EOCRC being associated with a single factor and instead study factors together using allostatic load. Although none of the papers measured AL and EOCRC directly, they each used a direct or indirect measurement of AL when studying EOCRC. We believe that AL should be the next focus for future research of EOCRC to better understand how all these factors contribute to disease progression.

## 1 Introduction

Colorectal cancer is the third most common cancer and accounts for ~10% of new cases per year. Although incidence rates of colorectal cancer have decreased in recent years, according to NIH Surveillance, Epidemiology, and End Results Program (SEER), it remains the third most deadly type of cancer and accounts for ~10% of cancer deaths annually.

Although colorectal cancer incidence rate has been on the decline, there is a split difference in trend between those who are older than 50 years and those who are younger than 50 (early-onset) (1). The decline in those >50 (LOCRC) has been attributed to increased screening and diagnostic tools, such as colonoscopy, as well as changes in lifestyle and diet. The rise in early-onset colorectal cancer (EOCRC) has been attributed to environmental and lifestyle factors that affect early life and young adulthood, including modern diet (1).

Given the current trend of EOCRC the annual percentage change-based predicted incidence rates of EOCRC will rise by over 90% by 2030 (2). Some evidence indicates that early-onset CRC is more likely to start out in the rectum compared to late-onset CRC. Those diagnosed with EOCRC are also more likely to be diagnosed with sporadic onset as opposed to those who carry the 5% germline mutation or those with a family history, representing 25% of overall cases (2).

The current research investigating EOCRC focuses mostly on epidemiology, pathology, or individual risk factors, emphasizing singular causes rather than multifactorial contributions. While these studies have provided valuable insights into early-onset cancer, there is still a need to examine each factor in relation to one another rather than separately.

An alternative mechanism for evaluating EOCRC is allostatic load. Allostatic load, as defined by McEwen and Stellar, is the body’s adaptation to chronic stress over time (3). This conceptual and biomarker-based framework emphasizes the relationship between environmental factors and genetic predispositions that make some individuals more susceptible to disease. The body’s first response to stress activates the neuroendocrine pathways, which secrete norepinephrine, epinephrine, cortisol, and dehydroepiandrosterone sulfate (DHEA-S) (4). Also, in conjunction with neuroendocrine signaling, pro- and anti-inflammatory cytokines (e.g., interleukin-6 and tumor necrosis factor-alpha) are released in response to stress. These represent primary mediators of AL biomarkers. Over time, chronic stress can impact other biological pathways and secondary mediators, such as cardiovascular (systolic and diastolic blood pressure) and metabolic (cholesterol levels, HbA1c, waist circumference, triglycerides, high-density lipoprotein (HDL), and low-density lipoprotein (LDL)). The impact of secondary mediators leads to physiological dysregulation, thereby increasing cancer susceptibility (4).

A study by Geronimus et al. further examined the link between allostatic load and race, coining the term “weathering” (5). Weathering considers how the cumulative impact of chronic exposure to socioeconomic stress and political marginalization increases physiological deterioration, which leads to an increased susceptibility to diseases in African Americans in comparison to their Caucasian American counterparts. By connecting allostatic load with socioeconomic stress and political marginalization, Geronimus et al. provided context and a different paradigm for the current health outlook: health is not determined by a single factor but is multifactorial.

The purpose of this study is to apply a multifactorial lens to EOCRC. We hypothesize that there are direct and indirect links between AL and EOCRC. In this paper, we use AL as a framework to explore how chronic stress-related physiological dysregulation may intersect with diet and lifestyle factors, epigenetic modifications, and microbiome alterations that have been implicated in EOCRC.

## 2 Methods

### 2.1 Study protocol

This paper aims to review high-quality research that assesses allostatic load and EOCRC. We adhered to the guidelines set forth by the “Preferred Reporting Items for Systematic reviews and Meta-Analyses” (PRISMA) framework. The paper includes a comprehensive search of both cohort and case-control studies that were designed to investigate the association between EOCRC and allostatic load (AL).

### 2.2 Search strategies

**Figure 1:**
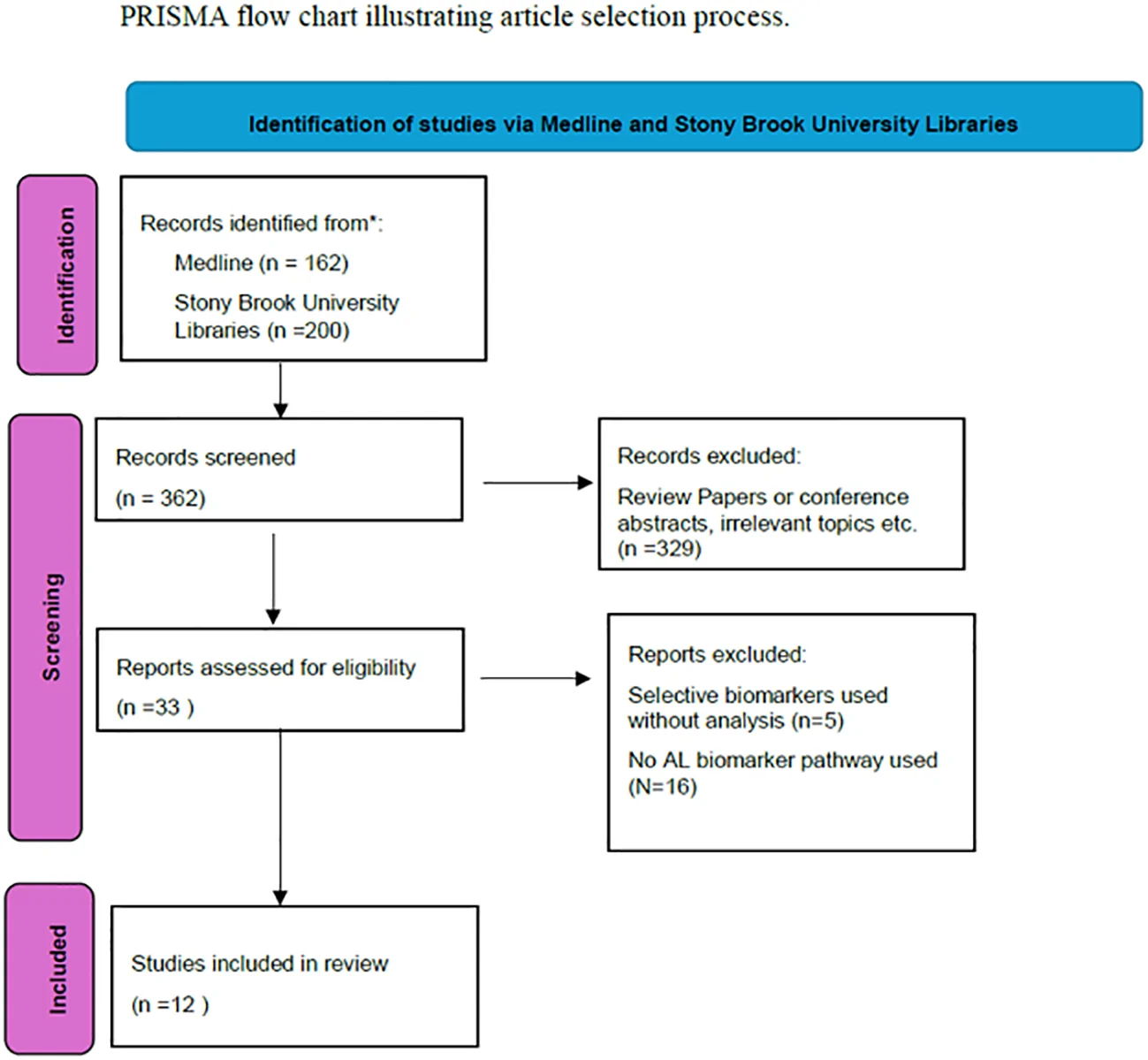
PRISMA flow chart illustrating article selection process.

The investigators performed a comprehensive literature search using 2 databases: MEDLINE PubMed, and the Stony Brook University Libraries Database as shown in **Figure 1**. The search was done separately for each topic: lifestyle, microbiome, and epigenetics. Searches for lifestyle were conducted using the existing advanced search options in the Stony Brook University Libraries Database. The specific search used, “Colorectal Cancer AND (Early onset OR young onset),” produced upwards of 200 initial studies. Medline was used to identify papers that include the terms “microbiome” and “early-onset colorectal cancer” (n = 51) as well as “microbiome” and “young-onset colorectal cancer” (n = 11). To identify peer-reviewed literature investigating the intersection of allostatic load, epigenetic modifications, and early-onset cancers, we conducted a systematic search of the PubMed/MEDLINE database. Our search strategy integrated official Medical Subject Headings (MeSH) with targeted free-text keywords, employing nested Boolean logic to bridge molecular mechanisms, social determinants, and clinical pathology. Specifically, we used the search string: [“Epigenomics”(Mesh) OR “Early Onset Colorectal Neoplasms”(Mesh)]. Following the initial search, there were over 100 papers. We refined the results by applying PubMed’s standardized age filters to isolate “early-onset” populations.

### 2.3 Eligibility and screening

All topics were screened to exclude review articles, preprints, non-human studies, and papers that did not meaningfully focus on EOCRC in their analyses. Studies that only focused on CRC and did not include EOCRC were not included. Studies were eligible for selection if there was a clear indication of relevance to at least one widely accepted AL marker. Such markers included: BMI, cortisol, triglycerides (TyG), blood pressure (systolic or diastolic), HbA1c, waist circumference (WC), and total cholesterol (TC) (5). We also included papers that indirectly referenced biomarkers through either cardiovascular, neuroendocrine, inflammatory or metabolic pathways. Preference was given to studies with quantitative analysis of AL markers. This yielded 12 eligible studies published between 2019 and 2026.

### 2.2 Quality assessment

The studies were assessed by three authors (EE, SW, and JC) using the Newcastle-Ottawa Quality Assessment Form which assessed for cross-sectional and cohort studies. Based on the Newcastle-Ottawa scales (NOS), studies were interpreted as: Good = 7–9 stars; Fair = 5–6 stars; Poor = 0–4 stars. The two authors independently reviewed each based on the NOS criteria. Where there was a difference in score, a majority rules was applied to avoid discordance.

## 3 Results

### 3.1 Search results

The systematic search identified 362 articles, and only 33 were eligible for full-text screening. After full-text screening, 12 articles were included for the final synthesis.

### 3.2 Study quality

Of 12 studies, 7 received a rating of “Good” as seen in **Table 1**. Across all 12 studies, they clearly specified their objectives, population, and exposures to study EOCRC and their biomarker of focus. However, some methodologies were often ignored such as follow-ups for cohort studies or providing non-response rates in case control-studies.

**Table 1:** Study characteristics and allostatic load pathway.

First author (year)	category	Metabolic	Inflammatory	Neuroendocrine	Cardiovascular	Study design	Sample size	Study quality
[Low (6)]	lifestyle	BMI	N/A	N/A	N/A	Conducted a case-control study of US veterans 18 to 49 years old who underwent colonoscopy examinations from 1999 through 2014. EOCRC cases were identified from a national cancer registry; veterans who were free of CRC at their baseline colonoscopy through 3 years of follow-up were identified as controls. Collected data on age, sex, race/ethnicity, body weight, body mass index (BMI), diabetes, smoking status, and aspirin use.	68,067 veterans under the age of 50 who had undergone colonoscopy between 1999 and 2014, of which 651 were considered “case selections”	Good
[Kim (7)]	lifestyle	BMI, WC,FBG, TyG, HDL	N/A	N/A	BP	Performed a cross sectional analusis on medical history and health-related behavior were collected from self-administered questionnaires. Patients were divided into groups based on age (20–29 years, n = 7340 or 30–39 years, n = 65,016), and χ 2 tests were used to compare categorical variables between groups.	72,356 asymptomatic individuals, 20–39 years old, who underwent colonoscopies as participants in the Kangbuk Samsung Health Study in South Korea, from August 2004 through December 2015.	Good
[Jin (8)]	Lifestyle	BMI, WC, TC, TyG, HDL, FBG	N/A	N/A	BP	Collected data on age, sex, lifestyle factors, body mass index (BMI), waist circumference (WC), blood pressure, and laboratory findings. A multivariate Cox proportional hazards regression analysis was performed.	Nationwide population-based cohort study enrolled 9,774,081 individuals who underwent health checkups under the Korean National Health Insurance Service from 2009 to 2010, with follow-up until 2019.	Good
[Li (9)]	Epigenetics	APOL3, MFAP2	N/A	N/A		Bioinformatics experimental study using TCGA-COAD datasets from the public repository	9 EOCRC Tumors and 7 Matched Normal Adjacent Tissue (NAT) samples were used in this study	Fair
[Yang (10)]	Microbiome	N/A	TLR, NF-κB	N/A	N/A	A multi-center observational case–control study using fecal microbiome profiling via 16S rRNA gene sequencing and shotgun metagenomic sequencing, combined with machine-learning–based (random forest) biomarker discovery and independent external validation cohorts. Through 16S rRNA gene sequencing, 728 samples were collected to identify microbial markers, and an independent cohort of 310 samples was used to validate the results.	A total of 1,038 participants were included, comprising 185 young-onset CRC cases, 379 old-onset CRC cases, and 474 age-matched healthy controls (217 age-matched healthy controls for the yCRC (yControl), and 257 age-matched healthy controls for the oCRC	Good
[Xiong (11)]	Microbiome	N/A	TLR, NF-κB	N/A	N/A	A single-center, cross-sectional case–control study using fecal 16S rRNA gene sequencing, with taxonomic and functional predictions (COG and KEGG) to compare microbiota composition among EOCRC patients, late-onset CRC patients, and healthy young controls.	The study included 98 participants total: 24 EOCRC patients, 43 late-onset CRC patients, and 31 healthy young controls.	Fair
[Kong (12)]	Microbiome	N/A	TLR	N/A	N/A	Discovery cohort included 114 early-onset CRC (EO-CRC), 130 late-onset CRC (LO-CRC), and matched controls (100 EO, 97 LO); validated in an independent cohort with 24 EO-CRC, 38 LO-CRC, and matched controls.	Discovery cohort included 114 early-onset CRC (EO-CRC), 130 late-onset CRC (LO-CRC), and matched controls (100 EO, 97 LO); validated in an independent cohort with 24 EO-CRC, 38 LO-CRC, and matched controls.	Good
[Adnan (13)]	Microbiome	N/A	NF-κB	N/A	N/A	A bioinformatics-driven observational study integrating fecal shotgun metagenomic data from multiple international cohorts (CuratedMetagenomeData) with tumor-associated microbiome and host gene expression data from TCGA-COAD, using multivariable regression and pathway-level correlation analyses to assess age-specific effects.	The fecal microbiome analysis included 1,394 individuals (701 CRC cases and 693 controls), while tumor-associated microbiome and host–gene interaction analyses were conducted in a separate dataset of 85 CRC patients (15 early-onset and 70 late-onset).	Good
[Barot (14)]	Microbiome	N/A	TLR, NF-κB	N/A	N/A	A prospective comparative cohort study comparing young-onset colorectal cancer with average-onset colorectal cancer. Fresh frozen tumor tissue and paired adjacent non-malignant tissue were collected from each patient. Microbial profiles were analyzed using 16S rRNA amplicon sequencing and bioinformatics tools to compare diversity, microbial abundance, clinical associations, and survival outcomes.	The study included a total of 276 colorectal cancer patients. Of these, 136 patients had young-onset colorectal cancer (yoCRC, age <50) and 140 had average-onset colorectal cancer (aoCRC, age >60). Each patient provided both tumor and paired adjacent non-malignant tissue specimens for analysis.	Fair
[Qin (15)]	Microbiome	N/A	TLR	N/A	N/A	Multicohort shotgun metagenomic analysis combining a newly generated hospital cohort, a four-group age-structured cohort, and eight publicly available CRC cohorts reprocessed uniformly	~981 CRC tumors and ≥662 controls across all integrated datasets, including 72 EOCRC cases (<50 yrs).	Good
[Jayakrishnan (16)]	Microbiome	N/A	TLR, NF-κB	N/A	N/A	A comparative, observational multi-omics study integrating tumor microbiome sequencing (16S rRNA) and plasma metabolomics from CRC patients stratified by age	64 CRC patients (20 EOCRC ≤50; 44 older ≥60). Each patient contributed tumor tissue for microbiome analysis and plasma samples for metabolomics profiling.	Fair
[Deng (17)]	Microbiome	N/A	TLR, NF-κB	N/A	N/A	Observational study with 16S and shotgun metagenomics; mediation + logistic regression for prediction.	76 microbiome samples (discovery/validation) + 298 participants for lifestyle/diet validation.	Good

BMI, Body Mass Index; WC, Waist Circumference; TC, Total Cholesterol; TyG, Triglyceride; HDL, High density lipoprotein; FBG, Fasting Blood Glucose; APOL3, Apolipoprotein.

### 3.3 Study results

The 12 articles selected (**Figure 1**) for this review were published between 2019 and 2026. Each study varied by geographic location, with 2 in South Korea (7, 8), 5 in China (10–12, 15, 17) and 5 in the United States of America (9, 13, 14, 16), and (6). Due to the field’s novelty, not every study included or mentioned standard biomarkers. The studies were divided into risk factors (lifestyle, microbiome, and epigenetics) and allostatic load categorical pathways (cardiovascular, neuroendocrine, inflammatory, or metabolomics) divided into direct pathways. Direct measurements of AL include the current standard biomarkers, while indirect measurements include biomarkers that can affect direct measurements of AL, as shown in **Figure 1**.

Of the 12 studies examined only 3 had direct measurements of AL. These included metabolic and cardiovascular, although cardiovascular measurements were provided in conjunction with metabolic syndrome. 1 Paper had an indirect measurement of metabolic syndrome, using genes to showcase differences in race and EOCRC. 8 papers indirectly measured inflammatory pathways studying the microbiome in those with EOCRC compared with those with LOCRC. No papers studied examined the relationship between neuroendocrine pathways and EOCRC.

### 3.4 Direct allostatic load measurements and EOCRC

#### 3.4.1 Lifestyle

Of the 12 studies examined, 3 were found to be related to lifestyle factors commonly associated with CRC and explored how various aspects of said lifestyle factors contributed to EOCRC. Of these, one study examined the effect of various lifestyle factors as risk indicators for EOCRC. Obesity, smoking status, aspirin use, and diagnoses of diabetes were the main risk factors considered that could be classified as lifestyle factors. Also considered for study inclusion were race, age, and sex.

All 3 studies reported BMI measurements, as it is associated with EOCRC (6–8). Two studies reported that an increase in BMI correlated with an increased risk of EOCRC, while one paper reported on increased risk of EOCRC with lower BMI. These conflicting reports may be due to differences in the timing of BMI measurement before colonoscopy and neoplasm detection.

Metabolic syndrome (MeS) was also highly associated with increased risk of EOCRC. According to the NHIS, metabolic syndrome is measured using waist circumference, and various AL biomarkers such as FBG, TyG, and HDL-C; and having high levels for 3/5 of these meets the criteria for metabolic syndrome. The cutoff for associating WC and abdominal obesity is >80 cm in women and >90 cm in men. Jin et al. reported that women with WC> 95 and men with WC >100 had a 53% increased risk of EOCRC compared with their controls. Elevated levels of FBG, TyG, BP, and HDL-C were significantly associated with increased risk of EOCRC in 2 papers (7, 8). Elevated levels measured for MeS included FBG>100mg/dL, TyG >150mg/dL, HDL-C <40mg/dL in men or <50mg/dL in women, and BP >140/85 (7, 8).

### 3.5 Indirect measurement of allostatic load and EOCRC

Out of the 12 studies examined, 9 did not directly link AL and EOCRC but they did study indirect pathways of AL that can contribute to EOCRC. The indirect pathways used were metabolic and inflammatory. No other pathways were examined in these studies.

#### 3.5.2 Microbiome

Of the studies examined, 8 directly evaluated the role of gut microbiome composition and function in early-onset colorectal cancer (EOCRC) (10–17). These studies compared patients under age 50 with either late-onset CRC patients or age-matched healthy controls and, in several cases, incorporated metagenomics, metabolomics, or machine-learning approaches to characterize taxonomic and functional differences (10–17). Although these papers do not measure a specific AL biomarker currently used, they instead assess inflammatory pathways.

The inflammatory biomarkers we considered were NF-κB and toll-like receptors (TLRs). Other pathways such as cardiovascular, metabolic, and neuroendocrine were not measured in these studies. Multiple papers on the microbiome and EOCRC found an increase in dysbiosis, with *Flavonifractor plautii, Fusobacterium nucleatum, Bacteroides fragilis, Akkermansia muciniphila, and Peptostreptococcus* being upregulated in EOCRC. *Flavonifractor plautii* and *Akkermansia muciniphila* were the two most common bacteria upregulated in EOCRC and these bacteria correlated with an increase in NF-κB, TLR2 and TL4, suggesting that these bacteria are proinflammatory and potentially increase the risk of EOCRC.

Across studies, EOCRC is marked by a shift in gut microbial metabolic function, with enrichment of taxa that participate in anaerobic fermentation, amino-acid metabolism, bile-acid transformation, and flavonoid and aromatic-compound degradation, alongside altered short-chain fatty acid (SCFA) pathways (10–12, 15). Recurrent enrichment of *Flavonifractor plautii* (10–12, 15, 17), *Fusobacterium nucleatum* (10, 14, 16, 17), Bacteroides fragilis (11, 13, 14), *Akkermansia muciniphila* (11, 13, 14), and *Peptostreptococcu*s (16, 17) species suggests that EOCRC-associated dysbiosis is driven by altered microbial metabolism rather than simple loss of protective taxa. Importantly, several studies report EOCRC-specific metabolic signatures. These signatures include increased flavonoid degradation, tryptophan metabolism, bile-acid metabolism, and choline utilization, indicating that microbial metabolic activity in younger patients may respond differently to dietary substrates and host-derived compounds compared with late-onset CRC (10, 12, 17).

#### 3.5.2 Epigenetics

Only one paper reported a connection between epigenetics and EOCRC with focusing on Black and non-white Hispanic people (9). Although AL biomarkers were not measured, genes associated with metabolic pathways were highlighted. Li et al. assessed the impact of allostatic load on early-onset colorectal cancer among people of different racial/ethnic backgrounds. The two genes found to be hypermethylated in Black and Hispanic samples were the apolipoprotein-encoding gene (APOL3), which is associated with lipid cholesterol metabolism, and MFAP2, which is associated with obesity and type 2 diabetes in humans.

## 4 Discussion

This paper is hypothesis-driven and aims to highlight a potential new framework to study the rise of EOCRC. Despite the abundance of reviews and articles on EOCRC, we found only 3 articles that directly measure allostatic load biomarkers and EOCRC, and 9 that indirectly measured these markers by looking at AL pathways. All articles were published within the past 7 years; from 2019 to 2026.

Previous studies have looked at allostatic load and cancer. Mathew et al. reported finding 12 papers that used allostatic load biomarkers and that there was a 9% increased risk of cancer mortality in those with an increase in allostatic load score (18). To delve deeper, breast cancer and AL have been studied, and it has been shown that an increase in allostatic load score is associated with an increase in mortality rate (19). Additionally, when evaluating AL and CRC, Zhao et al. used tumors from a UK biobank and found that, on average, those with higher AL scores had 40% increased risk of developing colon and rectal cancers. Their findings also indicate an association between high AL and elevated risk of CRC in those younger than 65 years of age (20). Studies have indicated that there is an association between adverse family environments, childhood socioeconomic status, adverse childhood experience and elevated AL levels (20). This highlights the need for further research on the impact of AL on EOCRC to develop a more accurate timeline for early intervention.

Our findings indicate that multiple factors may contribute to EOCRC, and that these factors should not be siloed. Because allostatic load is still a novel concept, few articles report findings that include allostatic load biomarkers. Allostatic load is measured in many ways, and the common method assigns each biomarker 1 point if the biomarker was indicated as being high risk (21). This is because measuring all the biomarkers may be tedious or unavailable. Currently, papers that included a direct measurement of allostatic load differ in the type of biomarkers they used. Of the 12 papers screened, only 3 measured allostatic load biomarkers, and the most common measurement was BMI, followed by waist circumference ratio, fasting blood glucose (FBG), triglycerides (TyG), high density lipoprotein (HDL), and blood pressure (BP) (6–8). Although 3 papers measured BMI, 2 concluded that higher BMI increases the risk of EOCRC, whereas Low et al. found the opposite. This discrepancy, as observed in Low et al., who measured weight immediately before colonoscopy, where patients were losing weight due to the cancer itself. These inconsistent findings are another reason why standardized measurements must be implemented so that we can better analyze such inconsistencies.

For indirect measurements, we examined the various biological pathways of interest: metabolic, neuroendocrine, inflammatory, and cardiovascular. For example, papers were more likely to include direct measurements of metabolic or cardiovascular pathways than of neuroendocrine or inflammatory pathways. These metabolic alterations closely intersect with host inflammatory signaling pathways, highlighting inflammation as a key mechanistic link between microbiome function and EOCRC. Across multiple cohorts, microbial metabolites and surface molecules were shown to modulate NF-κB and Toll-like receptors (TLR2 and TLR4) signaling pathways in intestinal epithelial and immune cells (10, 11, 14). Deng et al. studied the relationship between lifestyle, diet, and microbiome in EOCRC and found that those with increased alcoholic intake and red meat intake had increased dysbiosis and EOCRC. The paper from Deng et al. is one of the few papers regarding EOCRC, that proposes a connection between lifestyle and one’s microbiome.

Together, these findings may indicate that EOCRC is characterized by a metabolically active microbiome that drives context-dependent inflammatory responses, potentially creating a pro-tumorigenic microenvironment. The consistent involvement of diet-responsive metabolic pathways further underscores the relevance of lifestyle factors in shaping inflammatory risk in EOCRC (17). More specifically, mediation analyses indicated that pks^+^ E. coli (a strain of E. coli carrying the *pks* genomic island that promotes CRC) partially mediated the associations between fried foods, processed meat, and coffee consumption and ultimately EOCRC risk, whereas F. nucleatum mediated the detrimental effects of snack consumption (17). Microbial activity appears to function as a central link between lifestyle exposures, inflammatory processes, and the development of earlyonset colorectal cancer. This integrated model points toward the potential value of microbiomeguided prevention, risk assessment, and early detection strategies.

Although the microbiome and lifestyle are assessed in EOCRC studies, epigenetics remains unexplored. Allostatic load can alter epigenetics and, as such, DNA methylation patterns are among the proposed causes of CRC (9). Although Li et al. did not specifically study allostatic load and epigenetics in EOCRC, data indicated that epigenetic changes in Black and non-white Hispanic EOCRC’s tumors differ from their white counterparts. The gene MFAP2 was shown to be hypermethylated in minorities (9). Thus, when considered in conjuction, these data indicate that lived experiences affect epigenetic mechanisms and can increase the risk of EOCRC. Given MFAP2’s role in metabolism and disease, this methylation pattern might serve as a potential biomarker for EOCRC.

Lived experiences, in relation to Geronimus’ “weathering”, illustrates that multiple stressors can affect changes even at the biological level, which can make people susceptible to cancer. But the question remains: why is there an increase in EOCRC in comparison to LOCRC if wear and tear happens with age? One possibility is the change in diet and lifestyle over time. Over the past two centuries the American diet has seen an increase in consumption of ultra-processed food (sugar, white flour, and rice) and poultry and reduced consumption of unprocessed foods (fresh fruits and vegetable) (22). A diet rich in fats and sugar also known as an inflammatory diet may damage the intestinal microenvironment and the inflammation in the gut can trigger hyperplasia and polyp growth leading to CRC (23). Sedentary lifestyle has also increased in the US for the past 50 years with more access to modern technology and the increased use and reliance of cars (24). The combination of sedentary lifestyle and poor diet has been associated with increases in CRC and may help to explain the noted increase in EOCRC (25). Another possibility is increase in cumulative stress. General studies have shown that stress can exacerbate aging which leads to disease (26, 27). Alongside changes in diet and lifestyle, stress has also seen an increase in young adults. In general, over the past 20 years, younger adults have been shown to have the highest levels of stress and stress reactivity compared to other age groups (28). Stress has been shown to shorten telomere length which leads to premature aging (29).

Another method to study premature aging is using epigenetic clocks. Epigenetic clocks are DNA methylation markers that correlate with chronological age and can serve as a molecular estimator of biological age in cells, tissues, and individuals (30). With younger adults suffering more from stress, and having negative affect reactivity, this can accelerate the wear and tear process seen in normal aging and cause premature illness. General aging and stress data have shown that chronic stress has been linked with obesity and lead to perturbation to the hypothalamic pituitary adrenal (HPA) axis (31, 32). Taken together, chronic stress and the modern western diet may cause epigenetic changes leading to premature aging and an increase risk of EOCRC.

## 5 Conclusion and future directions

Although the evidence discussed is preliminary, this piece highlights the need to study risk factors and how these factors interact to contribute to EOCRC. The purpose of this paper is to provide an alternative mechanism to study EOCRC. Allostatic load can potentially be used to study this phenomenon because it also adds context to the data. Factors such as environment, socioeconomic status, diet, and access to healthcare can possibly impact people on a cellular level and thus potentially influence biological changes that may impact one’s susceptibility to cancer. The current paradigm focuses on different potential causes of EOCRC; what we hope to change is combining these different metrics to study EOCRC using AL. To truly understand how allostatic load can be used to study increased risk in EOCRC, there must be standardized measurements of biomarkers and data collection reports. This would ensure better established baselines for EOCRC. Instead of data being released as dichotomous variables, continuous variables will allow a better understanding of the degree to which these biomarkers are associated with increased risk of EOCRC. For example, a person can have high blood pressure but does having 140/80 impact the risk more or less than with a pressure of 210/100? These intricacies are important to know what cutoff values places a person more at risk. Having data on all biomarkers can allow for more in-depth analysis on the impact of allostatic load on the risk of EOCRC. This paradigm shift to focus on the combined impact of each risk factor together, rather than in isolation, could allow for better understanding of the causes of EOCRC.

## 6 Limitations

This paper has many limitations. Only a small number of papers reported direct links to AL (n=3). Due to the limited sample size, the study does not support causal relationship between AL and EOCRC. In addition, there were no longitudinal study about AL and EOCRC. The reported literature differs in the biomarkers used and in how they were measured. Some articles reported direct biomarker measurements, while others only presented odds ratios of measured values. These inconsistencies made it difficult to perform a meta-analysis to determine definitively how reported biomarkers may increase the risk of EOCRC. Another limitation is that not all studies included direct biomarker measurements; indirect measurements had to be used in their place. The papers that measured AL and EOCRC did not provide a score indicating the extent to which AL affects the risk of EOCRC. Without this score, it is difficult to determine conclusively whether higher AL is associated with EOCRC.

Due to the small sample size, the evidence connecting EOCRC and AL are preliminary and thus limitations highlights the need to further study and delineate the link of AL with EOCRC.

## Data Availability

The original contributions presented in the study are included in the article/supplementary material. Further inquiries can be directed to the corresponding author.

## References

[1] Sung H Siegel RL Laversanne M Jiang C Morgan E Zahwe M . Colorectal cancer incidence trends in younger versus older adults: an analysis of population-based cancer registry data. Lancet Oncol. (2025) 26:51–63. doi: 10.1016/S1470-2045(24)00600-4 39674189 PMC11695264

[2] Takada K Hotta K Kishida Y Ito S Imai K Ono H . Comprehensive analysis of early-onset colorectal cancer: a review. J Anus Rectum Colon. (2023) 7:241–9. doi: 10.23922/jarc.2023-032 37900694 PMC10600264

[3] McEwen BS Stellar E . Stress and the individual: mechanisms leading to disease. Arch Internal Med. (1993) 153:2093–101. doi: 10.1001/archinte.1993.00410180039004 8379800

[4] McEwen BS . Protective and damaging effects of stress mediators: central role of the brain. Dialogues Clin Neurosci. (2006) 8:367–81. doi: 10.31887/DCNS.2006.8.4/bmcewen 17290796 PMC3181832

[5] Geronimus AT Hicken M Keene D Bound J . Weathering" and age patterns of allostatic load scores among blacks and whites in the United States. Am J Public Health. (2006) 96:826–33. doi: 10.2105/ajph.2004.060749 16380565 PMC1470581

[6] Low EE Demb J Liu L Earles A Bustamante R Williams CD . Risk factors for early-onset colorectal cancer. Gastroenterology. (2020) 159:492–501.e497. doi: 10.1053/j.gastro.2020.01.004 31926997 PMC7343609

[7] Kim NH Jung YS Yang HJ Park SK Park JH Park DI . Prevalence of and risk factors for colorectal neoplasia in asymptomatic young adults (20-39 years old). Clin Gastroenterol Hepatol. (2019) 17:115–22. doi: 10.1016/j.cgh.2018.07.011 30025922

[8] Jin EH Han K Lee DH Shin CM Lim JH Choi YJ . Association between metabolic syndrome and the risk of colorectal cancer diagnosed before age 50 years according to tumor location. Gastroenterology. (2022) 163:637–648.e632. doi: 10.1053/j.gastro.2022.05.032 35643169

[9] Li JS Riggins K Yang L Chen C Castro P Alfarkh W . DNA methylation profiling at base-pair resolution reveals unique epigenetic features of early-onset colorectal cancer in underrepresented populations. Clin Epigenet. (2025) 17:11. doi: 10.1186/s13148-025-01817-z 39844333 PMC11753045

[10] Yang Y Du L Shi D Kong C Liu J Liu G . Dysbiosis of human gut microbiome in young-onset colorectal cancer. Nat Commun. (2021) 12:6757. doi: 10.1038/s41467-021-27112-y 34799562 PMC8604900

[11] Xiong H Wang J Chang Z Hu H Yuan Z Zhu Y . Gut microbiota display alternative profiles in patients with early-onset colorectal cancer [original research. Front Cell Infect Microbiol. (2022) 12. doi: 10.3389/fcimb.2022.1036946 36389150 PMC9648186

[12] Kong C Liang L Liu G Du L Yang Y Liu J . Integrated metagenomic and metabolomic analysis reveals distinct gut-microbiome-derived phenotypes in early-onset colorectal cancer. Gut. (2023) 72:1129–42. doi: 10.1136/gutjnl-2022-327156 35953094

[13] Adnan D Trinh JQ Sharma D Alsayid M Bishehsari F . Early-onset colon cancer shows a distinct intestinal microbiome and a host-microbe interaction. Cancer Prev Res (Phila). (2024) 17:29–38. doi: 10.1158/1940-6207.Capr-23-0091 37967575 PMC10842926

[14] Barot SV Sangwan N Nair KG Schmit SL Xiang S Kamath S . Distinct intratumoral microbiome of young-onset and average-onset colorectal cancer. EBioMedicine. (2024) 100:104980. doi: 10.1016/j.ebiom.2024.104980 38306898 PMC10850116

[15] Qin Y Tong X Mei WJ Cheng Y Zou Y Han K . Consistent signatures in the human gut microbiome of old- and young-onset colorectal cancer. Nat Commun. (2024) 15:3396. doi: 10.1038/s41467-024-47523-x 38649355 PMC11035630

[16] Jayakrishnan TT Sangwan N Barot SV Farha N Mariam A Xiang S . Multi-omics machine learning to study host-microbiome interactions in early-onset colorectal cancer. NPJ Precis Oncol. (2024) 8:146. doi: 10.1038/s41698-024-00647-1 39020083 PMC11255257

[17] Deng J-W Zhou Y-L Zhang Y-X Zhou C-B Fang J-Y . The relationship between gut microbiota, lifestyle habits, and early-onset colorectal cancer: shedding light on early prediction. Br J Cancer. (2026) 134:469–76. doi: 10.1038/s41416-025-03277-x 41366061 PMC12852739

[18] Mathew A Doorenbos AZ Li H Jang MK Park CG Bronas UG . Allostatic load in cancer: a systematic review and mini meta-analysis. Biol Res Nurs. (2021) 23:341–61. doi: 10.1177/1099800420969898 33138637 PMC8755951

[19] Dessie ZG Zewotir T . Global determinants of breast cancer mortality: a comprehensive meta-analysis of clinical, demographic, and lifestyle risk factors. BMC Public Health. (2025) 25:2640. doi: 10.1186/s12889-025-24036-w 40760691 PMC12323182

[20] Zhao J Xue E Zhou S Zhang M Sun J Tan Y . Allostatic load, genetic susceptibility, incidence risk, and all-cause mortality of colorectal cancer. JNCI: J Natl Cancer Inst. (2024) 117:134–43. doi: 10.1093/jnci/djae223 39271163

[21] Duong MT Bingham BA Aldana PC Chung ST Sumner AE . Variation in the calculation of allostatic load score: 21 examples from NHANES. J Racial Ethn Health Disparities. (2017) 4:455–61. doi: 10.1007/s40615-016-0246-8 27352114 PMC5195908

[22] Lee JH Duster M Roberts T Devinsky O . United States dietary trends since 1800: lack of association between saturated fatty acid consumption and non-communicable diseases. Front Nutr. (2021) 8:748847. doi: 10.3389/fnut.2021.748847 35118102 PMC8805510

[23] Ahmad Kendong SM Raja Ali RA Nawawi KNM Ahmad HF Mokhtar NM . Gut dysbiosis and intestinal barrier dysfunction: potential explanation for early-onset colorectal cancer. Front Cell Infect Microbiol. (2021) 11:744606. doi: 10.3389/fcimb.2021.744606 34966694 PMC8710575

[24] Ng SW Popkin BM . Time use and physical activity: a shift away from movement across the globe. Obes Rev. (2012) 13:659–80. doi: 10.1111/j.1467-789X.2011.00982.x 22694051 PMC3401184

[25] Liu PH Wu K Ng K Zauber AG Nguyen LH Song M . Association of obesity with risk of early-onset colorectal cancer among women. JAMA Oncol. (2019) 5:37–44. doi: 10.1001/jamaoncol.2018.4280 30326010 PMC6382547

[26] Lavretsky H Newhouse PA . Stress, inflammation, and aging. Am J Geriatr Psychiatry. (2012) 20:729–33. doi: 10.1097/JGP.0b013e31826573cf 22874577 PMC3428505

[27] Piazza JR Almeida DM Dmitrieva NO Klein LC . Frontiers in the use of biomarkers of health in research on stress and aging. J Gerontol B Psychol Sci Soc Sci. (2010) 65:513–25. doi: 10.1093/geronb/gbq049 20647348 PMC2920946

[28] Almeida DM Rush J Mogle J Piazza JR Cerino E Charles ST . Longitudinal change in daily stress across 20 years of adulthood: results from the national study of daily experiences. Dev Psychol. (2023) 59:515–23. doi: 10.1037/dev0001469 36174182 PMC9993073

[29] Harvanek ZM Fogelman N Xu K Sinha R . Psychological and biological resilience modulates the effects of stress on epigenetic aging. Transl Psychiatry. (2021) 11:601. doi: 10.1038/s41398-021-01735-7 34839356 PMC8627511

[30] Bell CG Lowe R Adams PD Baccarelli AA Beck S Bell JT . DNA methylation aging clocks: challenges and recommendations. Genome Biol. (2019) 20:249. doi: 10.1186/s13059-019-1824-y 31767039 PMC6876109

[31] Chao AM Jastreboff AM White MA Grilo CM Sinha R . Stress, cortisol, and other appetite-related hormones: prospective prediction of 6-month changes in food cravings and weight. Obes (Silver Spring). (2017) 25:713–20. doi: 10.1002/oby.21790 28349668 PMC5373497

[32] Sinha R . Role of addiction and stress neurobiology on food intake and obesity. Biol Psychol. (2018) 131:5–13. doi: 10.1016/j.biopsycho.2017.05.001 28479142 PMC6784832

